# Lower Eyelid and Cheek Reconstruction With the First Dorsal Metacarpal Artery Free Flap: A Case Report

**DOI:** 10.7759/cureus.98445

**Published:** 2025-12-04

**Authors:** Sergio Alejandro Díaz Hernández, Diana Vianney Díaz Avila, Diego López Gutiérrez, Rafael Reynoso Campo, Alfonso E Echeverria Desentis

**Affiliations:** 1 Plastic Surgery, Hospital General de México “Dr. Eduardo Liceaga”, Mexico City, MEX

**Keywords:** basal cell carcinoma (bcc), fdma flap, free microvascular flap, lower eyelid reconstruction, microsurgery

## Abstract

Reconstruction of full-thickness lower eyelid defects remains a complex challenge due to the simultaneous requirements of functional protection, dynamic closure, and aesthetic harmony. Various local and free flaps exist, but limitations such as donor-site morbidity, bulkiness, or poor color match persist. To our knowledge, based on a structured literature search of PubMed, Scopus, and Web of Science (database inception to November 2025), there are no previously published reports describing the use of the first dorsal metacarpal artery (FDMA) flap as a free flap for reconstruction of lower eyelid or adjacent malar defects. We report the case of a 61-year-old male with a 4 × 3 cm left lower eyelid lesion diagnosed as basal cell carcinoma, adenoid (adenocystic) variant. After Mohs micrographic surgery, the resulting 3.6 × 3.2 cm full-thickness defect was reconstructed using a free FDMA flap harvested from the dorsum of the hand. Microvascular anastomoses were performed with the superficial temporal vessels, and the donor site was resurfaced with a full-thickness skin graft. The flap remained viable throughout hospitalization with no ischemic complications. On postoperative day seven, flap integration and donor-site healing were satisfactory. At six months, the patient showed complete eyelid closure (lagophthalmos 0 mm), eyelid margin symmetry within 1 mm, and a palpebral aperture of 9 mm. Objective assessment demonstrated favorable Patient and Observer Scar Assessment Scale scores (observer, 11/60; patient, 13/60), and the FACE-Q Eye Module confirmed high satisfaction (appearance, 88/100; comfort, 92/100). The flap showed stable contour and excellent integration with adjacent tissues. This case demonstrates the feasibility of using the free FDMA flap for complex lower eyelid and malar reconstruction, offering thin, pliable tissue with favorable aesthetic and functional outcomes. As the first reported application of this flap in this anatomical region, it broadens the reconstructive options for periocular oncologic defects and supports further investigation through larger and longer-term studies.

## Introduction

Reconstruction of soft tissue defects in the facial region poses a significant challenge in reconstructive surgery, given the intricate anatomy, essential function, and high aesthetic demands inherent to this area. The etiology of such defects is varied; however, defects resulting from the resection of a basal cell carcinoma (BCC), the most common malignant skin neoplasm [1], need to be studied for the achievement of successful facial reconstruction, as well as to improve the quality of life of patients significantly by restoring their facial appearance, self-esteem, and capacity for social integration [2].

Given the limitations of conventional eyelid and midface reconstruction techniques in large or full-thickness defects, exploring alternative microsurgical options capable of providing thin, pliable, and well-vascularized tissue becomes essential to achieving both functional stability and aesthetic fidelity.

Within the spectrum of reconstructive options for complex facial defects, microvascular free flaps have gained prominence for their ability to provide vascularized, adaptable tissue from distant donor sites [3]. The choice of the ideal flap remains an area of active research, driven by the need to reduce donor-site morbidity, improve integration into the recipient bed, and achieve natural aesthetic results. The dorsum of the hand provides skin with color and texture closely matching that of the face, facilitating harmonious integration. Furthermore, the first dorsal metacarpal artery (FDMA) flap benefits from a consistent and dependable vascular pedicle, which reduces the risk of ischemic events and enhances the survival of the transferred tissue [4].

This case report documents the successful and innovative use of the free FDMA flap for reconstruction of a complex facial defect following Mohs resection of BCC. By providing a detailed description of the surgical approach, assessing functional and aesthetic outcomes, and reviewing the available literature, this study aims to expand current knowledge of its role in facial reconstruction, highlighting key advantages and specific considerations for optimal clinical application, particularly given the limited prior reports on this technique.

## Case presentation

A 61-year-old male from Mexico City, with chronic sun exposure and no significant comorbidities, noted a slow-growing mass on his left lower eyelid beginning in 2019. Initially presumed to be benign, the lesion gradually enlarged and changed in appearance, prompting an incisional biopsy in October 2024. Histopathologic analysis demonstrated BCC, adenoid (adenocystic) variant, characterized by basaloid epithelial nests with adenoid-like spaces and an infiltrative growth pattern. In January 2025, the patient was referred to the Plastic, Aesthetic, and Reconstructive Surgery Service of the Hospital General de México “Dr. Eduardo Liceaga” for definitive surgical management.

**Figure 1:**
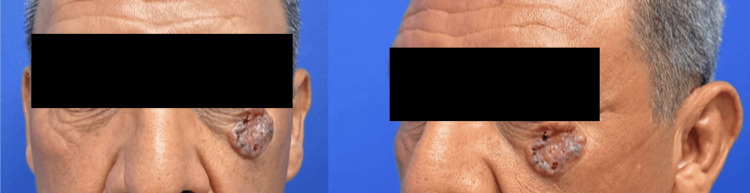
Preoperative clinical appearance of the lesion.

On preoperative examination, a 4 × 3 cm lesion was observed on the left lower eyelid, extending from the medial to the lateral canthal region. It exhibited irregular borders, peripheral hyperpigmentation, areas of superficial ulceration, and prominent telangiectasias (Figure 1). The patient subsequently underwent Mohs micrographic surgery performed by the Dermatology-Oncology Service, achieving tumor-free margins on frozen section analysis. The resultant defect measured 3.6 × 3.2 cm and required microsurgical soft-tissue reconstruction.

A free FDMA flap was designed on the dorsum of the right hand, incorporating the fascia of the first dorsal interosseous muscle. The flap was elevated on a 10-cm pedicle including the FDMA and two concomitant veins, providing sufficient length and caliber for microvascular transfer.

At the recipient site, the superficial temporal artery and its accompanying vein were dissected and prepared. End-to-end microvascular anastomoses were performed between the dorsal branch of the radial artery of the flap and the superficial temporal artery, and between one concomitant vein and a tributary of the superficial temporal vein. Arterial and venous diameters measured 1.0-1.2 mm at the recipient site and 0.8-1.0 mm at the flap pedicle. Mild size discrepancy was managed using beveling and gentle fish-mouthing of the smaller vessel to ensure adequate luminal match. Anastomoses were completed under 4.5× optical magnification using 10-0 nylon sutures for the artery and 11-0 nylon interrupted sutures for the vein, following standard microsurgical technique.

**Figure 2:**
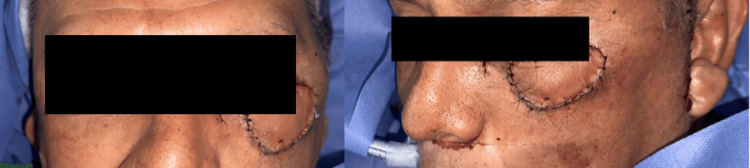
Post-Mohs defect and free first dorsal metacarpal artery flap inset.

**Figure 3:**
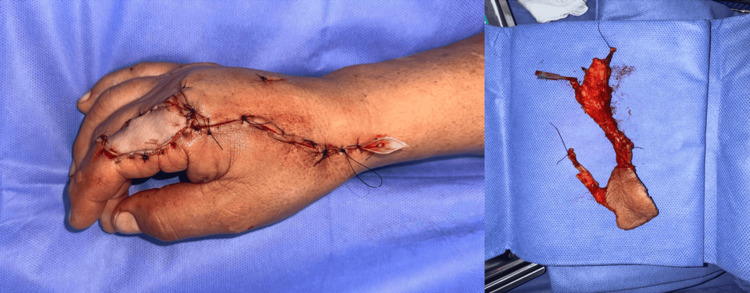
Donor site on the dorsum of the hand after flap harvest.

The skin island was inset into the lower eyelid defect with meticulous dermal coaptation and minimal tension to restore contour and mobility (Figure 2). The donor site was resurfaced with a full-thickness skin graft harvested from the ipsilateral arm (Figure 3). Perioperative anticoagulation consisted of heparinized saline flush (10 U/mL) intraoperatively, followed by aspirin 100 mg once daily for seven postoperative days, per institutional microsurgery protocol. Total operative time was approximately four hours, estimated blood loss was 300 mL, the surgical count was correct, and no intraoperative complications occurred. The patient tolerated the procedure well and was transferred to the recovery unit in stable condition.

In the immediate postoperative period, flap viability was assessed through a standardized microsurgical monitoring protocol. The patient was admitted to the postoperative care unit, where flap checks were performed hourly during the first 24 hours, then every 2-4 hours over the next 48-72 hours, and subsequently twice daily until discharge. Monitoring consisted of clinical assessment of color, temperature, turgor, capillary refill, and handheld Doppler signals over the arterial and venous pedicle. No implantable Doppler devices were required. Criteria for flap compromise included progressive pallor or congestion, loss of Doppler signal, delayed capillary refill (>3 seconds), or surface temperature drop >2°C compared to the surrounding skin. No signs of arterial or venous insufficiency were detected, and no re-exploration was necessary. The patient received prophylactic antibiotics and multimodal analgesia. He was discharged on postoperative day three with instructions regarding wound care and activity restrictions.

On postoperative day seven, suture removal revealed proper flap integration, satisfactory graft take at the donor site, and absence of infection, hematoma, or dehiscence. At the six-month follow-up, the patient demonstrated complete eyelid closure with a measured lagophthalmos of 0 mm, lower eyelid margin symmetry within 1 mm, and a vertical palpebral aperture of 9 mm compared to 10 mm in the contralateral eye. Eyelid function, including blink dynamics and orbicularis oculi strength, was evaluated using a standardized eyelid function assessment, showing normal blink excursion and full dynamic closure without retraction or ectropion.

**Figure 4:**
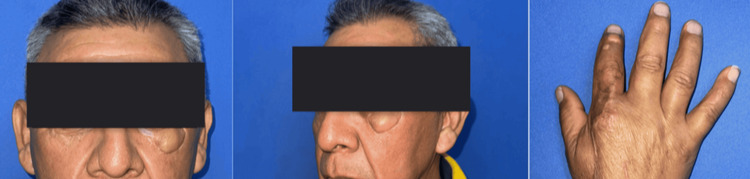
Six-month postoperative outcome of the recipient and donor sites.

Objective scar quality assessment was performed using the Patient and Observer Scar Assessment Scale (POSAS), resulting in a score of 11/60 (observer) and 13/60 (patient). Aesthetic and functional satisfaction were further evaluated with the FACE-Q Eye Module, where the patient reported high satisfaction with appearance (score: 88/100) and comfort (score: 92/100). Minimal scar contracture and excellent color and texture blending were observed clinically. The reconstruction remained stable and durable, with no episodes of flap compromise throughout follow-up (Figure 4, Table 1).

**Table 1:** Objective functional and aesthetic outcomes at the six-month follow-up. POSAS = Patient and Observer Scar Assessment Scale

Outcome domain	Measurement/Tool	Result	Notes
Eyelid closure	Lagophthalmos (mm)	0 mm	Complete closure
Eyelid symmetry	Margin position difference	1 mm	Compared to the contralateral side
Palpebral aperture	Vertical aperture	9 mm	Contralateral: 10 mm
Blink function	Functional assessment	Normal	Full dynamic closure, no ectropion
Scar quality	POSAS – Observer	11/60	Minimal scar contracture
	POSAS – Patient	13/60	Good patient-perceived scar quality
Aesthetic outcome	FACE-Q Eye Module – Appearance	88/100	High satisfaction
	FACE-Q Eye Module – Comfort	92/100	High satisfaction
Complications	Clinical evaluation	None	No ischemia, no re-exploration
Durability	Clinical evaluation	Stable reconstruction	No contour changes at 6 months

## Discussion

This case report describes the successful reconstruction of a left lower eyelid and adjacent malar region defect secondary to Mohs micrographic resection of a BCC using a free FDMA flap. The results demonstrate the feasibility and efficacy of this technique for restoring the function and aesthetics of the lower eyelid, with minimal donor-site morbidity and high patient satisfaction.

Following Mohs micrographic resection, a full-thickness eyelid defect was generated, involving approximately 70% of the left lower eyelid. Various reconstructive options were evaluated, including local flaps (forehead flap, Mustardé flap) and free flaps (radial forearm flap, temporoparietal fascia flap with skin graft). However, a free FDMA flap based on the first dorsal metacarpal artery was chosen for its inherent advantages. The skin on the dorsum of the hand presents ideal characteristics for eyelid reconstruction, such as thinness, flexibility, adaptability, and a similar texture and coloration to facial skin [5], which favors superior aesthetic integration and a more natural result compared to other, more voluminous free flaps or those with different qualities. In addition, the FDMA flap offers excellent vascular reliability and low donor-site morbidity [6].

The FDMA flap offers several advantages over other reconstructive options for eyelid defects. Its thinness and flexibility allow for optimal adaptation to the eyelid anatomy, avoiding the bulging and distortion that can occur with more voluminous flaps. Compared to other free flaps used in eyelid reconstruction, such as the radial forearm flap or the temporoparietal fascia flap, the FDMA flap presents significant advantages in terms of donor-site morbidity and surgical complexity. Primary closure of the donor site on the dorsum of the hand is feasible in most cases. It is associated with rapid healing and a low incidence of long-term complications. In contrast, the radial forearm flap can result in an unsightly scar and sensory disturbances in the forearm [7], while the temporoparietal fascia flap requires more extensive dissection and the placement of a skin graft on the scalp, which can cause permanent alopecia [8].

The scientific literature supports the use of the FDMA flap as a versatile and reliable reconstructive option. To our knowledge, there is no previously published report describing the use of a free FDMA flap for reconstruction of the lower eyelid and adjacent malar region. The only available reports of free FDMA flaps describe their application in other anatomical regions. For instance, Abu-Ghname et al. (2022) reported their experience with the free FDMA flap for reconstructing distal extremity defects in 16 patients, achieving a 95% survival rate [9]. To our knowledge, this is the first reported case of a free FDMA flap used for cheek reconstruction, thereby expanding the potential indications of this technique in complex facial defects.

These findings suggest opportunities to broaden the use of the FDMA-free flap for soft-tissue reconstruction, including in the facial region, as demonstrated in our case report. However, it is essential to note that most published studies are case reports or case series with a limited number of patients, which makes it difficult to generalize the results and directly compare them with other reconstructive techniques. Prospective, controlled studies with larger sample sizes and longer follow-up are needed to rigorously evaluate the benefits and limitations of the free FDMA flap across different clinical scenarios.

In the present case, the patient’s postoperative evaluation was conducted using a combination of objective and subjective tools. Flap viability was closely monitored; clinical evaluation demonstrated correct adaptation of the flap to the recipient bed, with adequate eyelid closure and minimal scar retraction. In addition, a patient satisfaction survey was administered, which revealed high satisfaction with the aesthetic and functional outcomes of the reconstruction.

This case report presents several limitations inherent to its design. First, it describes a single patient, which precludes generalizable conclusions regarding the effectiveness, safety, and reproducibility of the free FDMA flap for lower eyelid reconstruction. Future comparative studies could include prospective cohort analyses or randomized comparisons evaluating the FDMA flap against established reconstructive techniques such as the Mustardé cheek rotation flap, Tenzel semicircular flap, cervicofacial advancement flap, and free radial forearm flap, allowing assessment of relative advantages in contour, eyelid stability, donor-site morbidity, and complication rates.

Second, although clinical examination demonstrated satisfactory functional and aesthetic outcomes, more objective and standardized assessments would strengthen the findings. These include quantitative eyelid function measurements (lagophthalmos distance, margin reflex distance (MRD1 and MRD2), and palpebral aperture), validated functional scales such as the Eyelid Function Scale, and patient-reported outcome instruments such as the FACE-Q Eye Module, POSAS, or the National Eye Institute Visual Functioning Questionnaire-25 for vision-related quality of life.

Third, the postoperative follow-up period was limited to six months. This timeframe may not capture long-term complications such as flap thinning, contracture, or gradual contour changes. Future studies with extended follow-up and larger sample sizes would help clarify durability, aesthetic stability, and long-term donor-site outcomes.

Despite these limitations, this case contributes novel evidence supporting the feasibility of using the FDMA (Kite) flap as a free flap for lower eyelid reconstruction and establishes a foundation for future comparative and outcomes-based research.

## Conclusions

Lower eyelid reconstruction with the FDMA flap based on the first dorsal metacarpal artery is a valuable and reliable reconstructive option for the management of full-thickness defects following micrographic resection of BCC. The successful resolution of this case underscores the feasibility of this technique for simultaneously restoring the protective function and aesthetic harmony of the eyelid, minimizing donor-site morbidity, and maximizing patient satisfaction. Although the need for prospective, controlled studies with larger sample sizes and longer follow-up is acknowledged, the FDMA flap positions itself as an attractive, versatile option for facial reconstruction, offering an effective solution to a complex surgical challenge. Its potential to improve the quality of life of patients undergoing facial oncologic resections justifies further exploration and dissemination of this technique in the surgical community.
